# Risk factors and co-occurring patterns of low birth weight in Bangladesh: Insights from logistic regression and association rule mining

**DOI:** 10.1371/journal.pgph.0005177

**Published:** 2025-11-20

**Authors:** Md. A. Salam, Md. Merajul Islam, Md. Rezaul Karim

**Affiliations:** 1 Department of Statistics, University of Rajshahi, Rajshahi, Bangladesh; 2 Department of Statistics, Jatiya Kabi Kazi Nazrul Islam University, Mymensingh, Bangladesh; Aga Khan University, PAKISTAN

## Abstract

Low birth weight (LBW) remains a major public health concern in South Asia, including Bangladesh, contributing significantly to neonatal morbidity and mortality. This study aimed to identify individual risk factors for LBW using logistic regression (LR) and to explore co-occurring patterns among these risk factors through association rule mining (ARM). Analyzing the Bangladesh Demographic and Health Survey (BDHS), 2022 data with 1,435 participants, LR identified division, twin status, wealth index, place of delivery, duration of breastfeeding, and birth order as significant individual risk factors for LBW. The ARM revealed that infants in the Dhaka division with multiple births exhibited a higher risk of LBW, and this risk further increased when delivery occurred at a private facility. In Sylhet, LBW is more likely among 2^nd^ born children from low-wealth households who are not currently breastfeeding. In Chittagong, infants from single births who are not currently breastfeeding, delivered at home, and from low-wealth households are also at higher risk. Across all divisions, low-wealth households and lack of breastfeeding appeared as co-occurring patterns, indicating the combined influence of socioeconomic disadvantage and postnatal vulnerability among LBW infants. Combining LR and ARM provides a comprehensive understanding of individual and interacting LBW risk factors, supporting targeted interventions to lower LBW prevalence and neonatal mortality in Bangladesh, thereby contributing to SDG 3.

## 1. Introduction

Low birth weight (LBW) is a leading public health concern in developing nations, including Bangladesh [1,2]. LBW is defined as a birth weight of less than 2,500 grams [3]. Each year, approximately 30 million newborns, or 23.4% of all live births, are classified as underweight [4]. It is a prominent indicator contributing to neonatal morbidity and mortality, accounting for over 80% of newborn deaths globally [5,6]. The burden of LBW disproportionately affects low- and middle-income countries, with South Asia reporting the highest prevalence at 28%, followed by Sub-Saharan Africa at 13% [7,8]. In Bangladesh, the LBW was 17.7% in 2011 [9], 20% in 2014 [10], 16% in 2017 [11], and 14.5% in 2022 [12]. Although there is a decreasing trend, the rate remains high compared to other developing countries [13,14]. This underscores the urgent need to identify the risk factors contributing to LBW, implement effective policies, and adopt targeted interventions to address the underlying challenges that prevent better health outcomes for mothers and newborns.

Several studies in Bangladesh have investigated the risk factors of LBW using both traditional statistical methods and, more recently, machine learning (ML) algorithms. Islam et al. (2024) [15] analyzed nationally representative BDHS, 2017–18 data using multiple logistic regression (LR) to examine maternal, child, and household-level factors associated with LBW. They found that maternal age under 20 years, no formal education, female infant sex, poorest wealth quintile, and inadequate antenatal care (ANC) were significantly associated with higher odds of LBW. Furthermore, the study assessed the consequences of LBW on child health and nutrition, revealing that LBW children had a substantially higher risk of stunting, wasting, and underweight.

Tariqujjaman et al. (2024) [16] conducted a study to explore geographical disparities and socioeconomic inequalities in LBW prevalence and their association with maternal dietary diversity. They applied the concentration index (CI) to assess socioeconomic inequalities in LBW and performed a cluster-adjusted LR analysis to examine the association between LBW and maternal dietary diversity. They found that poor mothers (16%) contributed to having LBW babies, whereas the richest (10%) mothers gave birth to LBW babies. An adjusted LR identified that adolescent mothers with poor diets had the highest risk, 2.56 times more likely than older mothers with diverse diets.

Ahmed (2022) [17] utilized nationally representative MICS, 2019 data to map LBW prevalence across districts and identify socioeconomic predictors using LR. The analysis revealed an overall LBW prevalence of 14.5%, with the highest rates concentrated in the eastern as well as south-eastern districts of Bangladesh. Significant predictors included low maternal education, the poorest household wealth quintile, and urban residence. Alam et al. (2022) [18] investigated socioeconomic inequality in the prevalence of LBW among singleton births using BDHS data 2017–18. Applying LR and CI analysis, they found an overall LBW prevalence of 14.27%, with significantly higher rates among mothers from the poorest wealth quintile, those with no formal education, and those lacking improved toilet facilities.

Shaheen et al. (2020) [19] examined LBW prevalence and associated factors using retrospective hospital records from urban Dhaka (2014–2016). They used descriptive statistics, chi-square tests, and Pearson correlations to examine the relationships among LBW, maternal age, and socioeconomic status. Reza and Salma (2024) [20] analyzed BDHS, 2017–18 data, employing Boruta and wrapper-based feature selection methods to identify the important features of LBW. They applied both the traditional LR method and several ML algorithms, including decision trees (DT), support vector machine (SVM), Naive Bayes (NB), random forest (RF), extreme gradient boosting (XGB), and adaptive boosting (AdaBoost). This approach allowed a comparison of traditional and ML classifiers in predicting LBW.

Mansur et al. (2024) [21] adopted a unique multi-stage modelling framework using nationally representative MICS datasets from 2012–2013 and 2019, including district-level and national samples. They first used regression tree models to detect complex interactions among predictors. These interaction patterns were transformed into dummy variables and incorporated into fixed-effect, mixed-effect, and Besag-York-Mollie (BYM) spatial regression models, enabling both predictive modeling and spatial pattern analysis. Furthermore, the study investigated associations by extracting interpretable rules from regression trees, offering both accuracy and interpretability.

Islam et al. (2022) [22] also used BDHS 2017–18 data to predict LBW risk using LR and DT models. While they applied ML for classification, no association-rule mining was conducted. Across the literature, most of the studies have employed traditional LR to identify risk factors for LBW, while ML-based studies have focused on feature selection and predictive performance. However, only one study, Mansur et al. (2024) [21] applied association-rule extraction from regression trees for interpretable pattern discovery using MICS data.

No prior studies have combined LR and ARM to analyze risk factors for LBW using the most recent nationally representative BDHS data, 2022. Therefore, this study aimed to identify risk factors for LBW using LR and explore co-occurring patterns through ARM. The LR allows identification of individual risk factors, while ARM uncovers associations among co-occurring patterns/rules/synergetic clusters of these risk factors, providing a more comprehensive analytical approach. By revealing both individual and synergetic clusters of the risk factors, this study can inform targeted interventions and policy measures to reduce the prevalence of LBW, thereby enhancing health outcomes and supporting Sustainable Development Goal 3 (SDG-3), particularly target 3.2, which aims to mitigate neonatal mortality and address LBW by 2030 in Bangladesh.

The remainder of the paper is organized as follows: Section 2 outlines the materials and methods, including data source, variables, statistical analysis, logistic regression, and association rule mining. Section 3 presents the results, Section 4 discusses them, and Section 5 summarizes conclusions of the study.

## 2. Materials and methods

### 2.1. Data source

**Fig 1:**
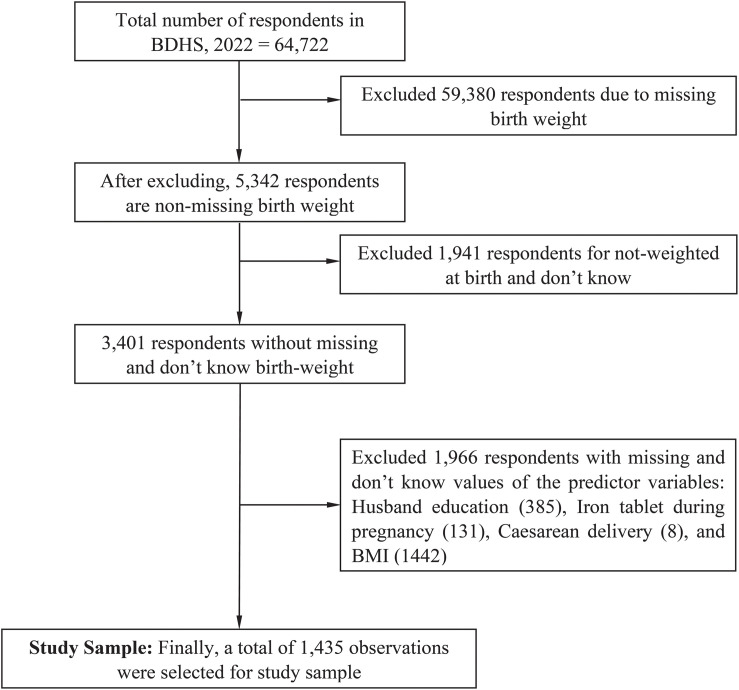
Study sample selection process.

This study used the most recent BDHS, 2022 data, conducted by the National Institute of Population Research and Training (NIPORT) in collaboration with ICF. The BDHS employed a two-stage stratified random sampling design [12]. In the 1^st^ stage, 675 enumeration areas were selected using probability proportional to size, including 438 rural and 227 urban. In the 2^nd^ stage, households were systematically selected within each enumeration area, totaling 30,375 households, including 19,710 rural and 10,665 urban. This approach ensured a representative sample of both urban and rural populations, providing comprehensive insights into Bangladesh’s health and demographic conditions. A total of 64,722 live-born infants were initially considered for this study, and after excluding records with missing, unknown, or irrelevant information, the final analytic sample comprised 1,435 infants with complete birth weight data. The sample selection process is illustrated in a CONSORT-style recruitment flowchart (Fig 1).

### 2.2. Ethical approval

The BDHS 2022 surveys (available on request at https://dhsprogram.com/data/dataset/Bangladesh_Standard-DHS_2022.cfm?flag=0) received ethical approval from the ICF Macro Institutional Review Board, Maryland, USA, and the National Research Ethics Committee of the Bangladesh Medical Research Council (BMRC), Dhaka, Bangladesh. This information on the ethics statement is provided in Lines 2–3 on Page 4 of the Bangladesh Demographic and Health Survey 2022 Key Indicators Report (given online at https://dhsprogram.com/pubs/pdf/PR148/PR148.pdf).

### 2.3. Outcome variable

The outcome variable in this study was LBW, defined according to the BDHS 2022 guideline [12]. According to the guideline, babies weighing less than 2,500 grams were classified as LBW (coded “1”), while those weighing 2,500 grams or more were classified as having normal birth weight (coded “0”). In the BDHS, 2022, data on birth weight were derived from written records or the mother’s report [12].

### 2.4. Explanatory variables

This study considered various explanatory variables, including demographic, socioeconomic, healthcare, and other factors, based on previous studies and the data available in the BDHS, 2022 database [23–32]. Demographic factors, include maternal age (15–19, 20–24, 25–29, 30–34, ≥ 35), division (Barisal, Chittagong, Dhaka, Khulna, Mymensingh, Rajshahi, Rangpur, Sylhet), residence (urban, rural), religion (muslim, non-muslim), sex of household head (male, female), sex of child (male, female), age of respondent at 1^st^ birth (<18, 18–24, > 24), twin status (single birth, multiple birth), and total children ever born (1–3, 4–5, > 5). Socioeconomic factors include education (none, primary, secondary, higher), husband’s education (none, primary, secondary, higher), currently working (no, yes), wealth index (low, medium, high), household members (1–2, 3–4, ≥ 5), toilet facility (unimproved, improved), reading newspaper (not at all, < 1 week, ≥ 1 week), watching television (not at all, < 1 week, ≥ 1 week), owning a mobile phone (yes, no), internet use (never, yes (last 12 month), yes (before last 12 months)), health insurance (no, yes); and Healthcare-related factors include body mass index (BMI) (underweight, normal, overweight, and obesity), age at first marriage (<18, 18–24, > 24), contraception use (no, yes), currently pregnant (no, yes), antenatal care visits (no, yes), iron tablet during pregnancy (no, yes) place of delivery (home, public, private, NGO/others), cesarean delivery (no, yes), duration of breastfeeding (not currently breastfeeding, never breastfeeding, still breastfeeding), wanted last child (wanted then, wanted later, wanted no more), birth order (1^st^, 2^nd^, 3^rd^ or more), and marriage to 1^st^ birth interval (<30, ≥ 30).

### 2.5. Statistical analysis

Descriptive statistics of the study participants were presented as frequencies and percentages. The chi-square test was used to assess the bivariate associations between the response and predictor variables, with a significance level set at p-value < 0.10 [33,34]. Subsequently, LR was then used to identify individual risk factors for LBW [35]. Odds ratios (ORs) with 95% confidence intervals (CIs) were calculated to assess the strength and direction of associations. Risk factors with *p* < 0.05 were considered statistically significant. Additionally, ARM was used to uncover co-occurring patterns or interactions of LBW risk factors [36]. ARM identifies relationships between risk factors in “if-then” rules (antecedent → consequent) and evaluates their strength using support, confidence, and lift. The Apriori algorithm was applied with a minimum support of 0.002 and a confidence threshold of 100%, enabling the identification of a reliable combination of risk factors contributing to childhood LBW [37]. Data analysis was performed using SPSS (version 27) and the R (version 4.4.2) programming language.

## 3. Results

### 3.1. Background characteristics of the study participants

**Fig 2:**
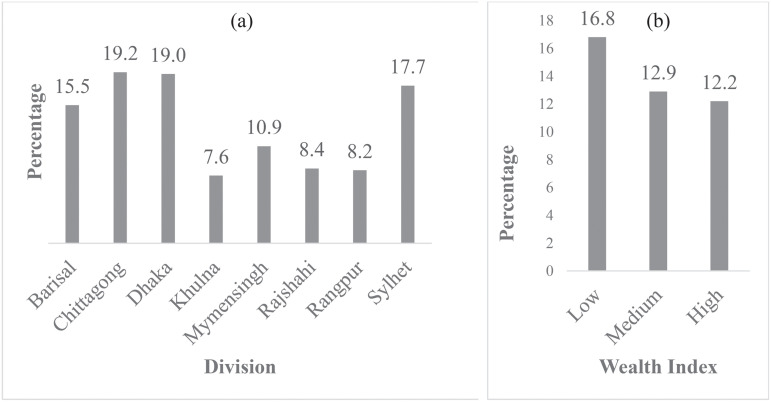
Low birth weight prevalence by geographic division and wealth index. (a) Division and (b) Wealth index.

Table 1 represents the background characteristics of the study participants. The highest percentage of children (17.3%) came from the Dhaka division, while the Sylhet division had the lowest percentage (9.1%). Most children (53.0%) had high socioeconomic status, while 27.5% had low socioeconomic status. About 67.2% of children were delivered to private institutions, 23.1% in public institutions, 7.0% at home, and 2.8% in NGO or other facilities. The overall prevalence of LBW is 13.6%. The prevalence of LBW across the eight geographic divisions of Bangladesh is presented in Fig 2a. The prevalence of LBW varies considerably between divisions. Chittagong (19.2%) and Dhaka (19.0%) report the highest LBW rates, closely followed by Sylhet (17.7%). In contrast, Khulna has the lowest prevalence (7.6%), with relatively low rates also observed in Rangpur (8.2%) and Rajshahi (8.4%). Mymensingh shows a moderate prevalence of 10.9%. Multiple births had a higher LBW prevalence (43.8%) compared to single births (13.2%), and their association is statistically significant. Fig 2b illustrates the prevalence of LBW by wealth index. LBW prevalence is highest among mothers in the low wealth index group (16.8%), followed by those in the medium wealth index group (12.9%), and lowest among the high wealth index group (12.2%).

**Table 1:** Background characteristics of the study respondents.

Predictor variable	Overall n (%)	LBW Status		p-value
		Yes, n (%)	No, n (%)	
**Overall**	1435 (100%)	195(13.6%)	1240(86.4%)	
**Demographic factors**				
**Maternal age**				
15-19	208(14.5)	32(15.4)	176(84.6)	0.821
20-24	494(34.4)	62(12.6)	432(87.4)	
25-29	398(27.7)	55(13.8)	343(86.2)	
30-34	233(16.2)	34(14.6)	199(85.4)	
≥35	102(7.1)	12(11.8)	90(88.2)	
**Division**				
Barisal	161(11.2)	25(15.5)	136(84.5)	<0.001
Chittagong	214(14.9)	41(19.2)	173(80.8)	
Dhaka	248(17.3)	47(19.0)	201(81.0)	
Khulna	210(14.6)	16(7.6)	194(92.4)	
Mymensingh	147(10.2)	6(10.9)	131(89.1	
Rajshahi	166(11.6)	14(8.4)	152(91.6)	
Rangpur	159(11.1)	13(8.2)	146(91.8)	
Sylhet	130(9.1)	23(17.7)	107(82.3)	
**Residence**				
Urban	537(37.4)	66(12.3)	471(87.7)	0.267
Rural	898(62.6)	129(14.4)	769(85.6)	
**Religion**				
Muslim	1288(89.8)	179(13.9)	1109(86.1)	0.312
Non-muslim	147(10.2)	16(10.9)	131(89.1)	
**Sex of household head**				
Male	1272(88.6)	167(13.1)	1105(86.9)	0.156
Female	163(11.4)	28(17.2)	135(82.8)	
**Sex of child**				
Male	771(53.7)	104(13.5)	667(86.5)	0.905
Female	664(46.3)	91(13.7)	573(86.3)	
**Age of respondent at 1**^**st**^ **birth**				
<18	386(26.9)	60(15.5)	326(84.5)	0.416
18-24	880(61.3)	114(13.0)	766(87.0)	
>24	169(11.8)	21(12.4)	148(87.6)	
**Twin status**				
Single birth	1419(98.9)	188(13.2)	1231(86.8)	0.002
Multiple birth	16(1.1)	7(43.8)	9(56.2)	
**Total children ever born**				
1-3	1358(94.6)	185(13.6)	1173(86.4)	0.745
4-5	75(5.2)	10(13.3)	65(86.7)	
>5	2(0.1)	0(0.0)	2(100.0)	
**Socioeconomic factors**				
**Education**				
None	22(1.5)	3(13.6)	19(86.4)	0.601
Primary	193(13.4)	25(13.0)	168(87.0)	
Secondary	792(55.2)	116(14.6)	676(85.4)	
Higher	428(29.8)	51(11.9)	377(88.1)	
**Husband education**				
None	22(1.5)	3(0.2)	19(1.3)	0.606
Primary	193(13.4	25(1.7)	168(11.7)	
Secondary	792(55.2)	116(8.1)	676(47.1)	
Higher	428(29.8)	51(3.6)	377(26.3)	
**Currently working**				
No	1159(80.8)	165(14.2)	994(85.8)	0.142
Yes	276(19.2)		246(89.1)	
**Wealth index**				
Low	394(27.5)	66(16.8)	328(83.2)	0.096
Medium	280(19.5	36(12.9)	244(87.1)	
High	761(53.0)	93(12.2)	668(87.8)	
**Household members**				
1-2	157(10.9)	25(15.9)	132(84.1)	0.386
3-4	857(59.7)	108(12.6)	749(87.4)	
≥5	421(29.3)	62(14.7)	359(85.3)	
**Toilet facility**				
Unimproved	335(23.3)	46(13.7)	289(86.3)	0.931
Improved	1100(76.7)	149(13.5)	951(86.5)	
**Reading Newspaper**				
Not at all	1272(88.6)	180(14.2)	1092(85.8)	0.176
<1 week	104(7.2)	11(10.6)	93(89.4)	
≥ 1 week	59(4.1)	4(6.8)	55(93.2)	
**Watching television**				
Not at all	560(39.0)	89(15.9)	471(84.1)	0.114
<1 week	120(8.4)	13(10.8)	107(89.2)	
≥ 1 week	755(52.6)	93(12.3)	662(87.7)	
**Owning a mobile phone**				
Yes	349(24.3)	50(3.5)	299(20.8)	0.644
No	1086(75.7)	145(10.1)	941(65.6)	
**Internet use**				
Never	843(58.7)	118(14.0)	725(86.0)	0.863
Yes (last 12 months)	584(40.7)	76(13.0)	508(87.0)	
Yes (before last 12 month)	8(0.6)	1(12.5)	7(87.5)	
**Health insurance**				
No	1429(99.6)	193(13.5)	1236(86.5)	0.414
Yes	6(0.4)	2(33.3)	4(66.7)	
**Health-related factors**				
**BMI**				
Underweight	177(12.3)	30(16.9)	147(83.1)	0.432
Normal	800(55.7)	109(13.6)	691(86.4)	
Overweight	371(25.9)	47(12.7)	324(87.3)	
Obesity	87(6.1)	9(10.3)	78(89.7)	
**Age at 1**^**st**^ **marriage**				
<18	819(57.1)	114(13.9)	705(86.1))	0.539
18-24	541(37.7)	74(13.7)	467(86.3)	
>24	75(5.2)	7(9.3)	68(90.7)	
**Contraception use**				
Yes	1029(71.7)	132(12.8)	897(87.2)	0.181
No	406(28.3)	63(15.5)	343(84.5)	
**Currently pregnant**				
No	1400(97.6)	188(13.4)	1212(86.6)	0.289
Yes	35(2.4)	7(20.0)	28(80.0)	
**Antenatal care visits**				
No	657(45.8)	93(14.2)	564(85.8)	0.565
Yes	778(54.2)	102(13.1)	676(86.9)	
**Iron tablet during pregnancy**				
No	187(13.0)	28(15.0)	159(85.0)	0.554
Yes	1248(87.0)	167(13.4)	1081(86.6)	
**Place of delivery**				
Home	100(7.0)	23(23.0)	77(77.0)	0.016
Public	331(23.1)	45(13.6)	286(86.4)	
Private	964(67.2)	125(13.0)	839(87.0)	
NGO/Others	40(2.8)	2(5.0)	38(95.0)	
**Delivery by cesarean**				
No	452(31.5)	74(16.4)	378(83.6)	0.037
Yes	983(68.5)	121(12.3)	862(87.7)	
**Duration of breastfeeding**				
Not currently breastfeeding	199(13.9)	42(21.1)	157(78.9)	0.001
Never breastfeeding	56(3.9)	11(19.6)	45(80.4)	
Still breastfeeding	1180(82.2)	142(12.0)	1038(88.0)	
**Wanted last child**				
Wanted then	1202(83.8)	161(13.4)	1041(86.6)	0.655
Wanted later	157(10.9)	21(13.4)	136(86.6)	
Wanted no more	76(5.3)	13(17.1)	63(82.9)	
**Birth order**				
1^st^	641(44.7)	72(11.2)	569(88.8)	0.065
2^nd^	522(36.4)	81(15.5)	441(84.5)	
3^rd^ or more	272(19.0)	42(15.4)	230(84.6)	
**Marriage to 1**^**st**^ **birth interval**				
<30	951(66.3)	134(14.1)	817(85.9)	0.437
>=30	484(33.7)	61(12.6)	423(87.4)	

The prevalence of LBW at home was highest (23.0%), followed by public facilities (13.6%), private facilities (13.0%), and NGO/other facilities (5.0%), and the association between these categories is statistically significant. Cesarean deliveries were significantly associated with lower LBW rates (12.3%) than normal deliveries (16.4%). Women who are not currently breastfeeding had a substantially higher LBW prevalence (21.1%) compared to those still breastfeeding (12.0%) and never breastfeeding (19.6%). The 2^nd^ and 3^rd^ babies showed greater prevalence (15.5%) and (15.4%), respectively, of LBW compared to the 1^st^ baby (11.2%).

### 3.2. Risk factors of LBW

We used LR analysis to identify the risk factors of LBW in Bangladesh, as shown in Table 2. The results indicated that children living in the Khulna (OR: 0.500, 95% CI: 0.254–0.983, p = 0.044) and Rangpur divisions (OR: 0.480, 95% CI: 0.233–0.989, p = 0.046) had lower odds of LBW compared to those in the Barisal division. Multiple births were more likely to be LBW than single births (OR: 4.464, 95% CI: 1.541–12.932). Children from high wealth index had significantly lower odds of LBW than those from low wealth index (OR: 0.601, 95% CI: 0.415–0.872, p = 0.007). Deliveries at public and NGO/other facilities were associated with lower odds of LBW compared to home deliveries (OR: 0.536, 95% CI: 0.294–0.978, p = 0.042), and (OR: 0.166, 95% CI: 0.036–0.757, p = 0.020), respectively. Babies who were still breastfeeding had lower odds of LBW compared to those not currently breastfeeding (OR: 0.552, 95% CI: 0.370–0.824, p = 0.004). 2^nd^ born babies had 1.53 times higher odds of having LBW than 1^st^ borns (OR: 1.530, 95% CI: 1.075–2.177, p = 0.018).

**Table 2:** Identification of risk factors of low birth weight using LR.

Predictor variables	AOR (95% CI)	p-value
**Division**		
Chittagong	1.292 (0.734, 2.277)	0.374
Dhaka	1.424 (0.821, 2.468)	0.208
Khulna	0.500 (0.254, 0.983)	0.044
Mymensingh	0.697 (0.352, 1.380)	0.301
Rajshahi	0.522 (0.257, 1.062)	0.073
Rangpur	0.480 (0.233, 0.989)	0.046
Sylhet	1.358 (0.714, 2.581)	0.351
Barisal^*^	1.000	
**Twin status**		
Multiple birth	4.464 (1.541, 12.932)	0.006
Single birth^*^	1.000	
**Wealth Index**		
Medium	0.715 (0.452, 1.131)	0.152
High	0.601 (0.415, 0.872)	0.007
Low^*^	1.000	
**Place of delivery**		
Public	0.536 (0.294, 0.978)	0.042
Private	0.603 (0.322, 1.128)	0.113
NGO/Others	0.166 (0.036, 0.757)	0.020
Home^*^	1.000	
**Cesarean delivery**		
Yes	0.868 (0.570, 1.323)	0.511
No^*^	1.000	
**Duration of breastfeeding**		
Never breastfeeding	0.825 (0.379, 1.796)	0.628
Still breastfeeding	0.552 (0.370, 0.824)	0.004
Not currently breastfeeding^*^	1.000	
**Birth order**		
2^nd^	1.530 (1.075, 2.177)	0.018
3^rd^ or more	1.146 (0.744, 1.767)	0.536
1^st*^	1.000	

*: Reference category.

### 3.3. Association rules for the likelihood of having LBW

This study used ARM to uncover synergetic clusters/patterns/rules among the LR-identified risk factors. A total of 32 rules with confidence ≥60% and significant lift values were found. Six of these rules had confidence over 100%, with the highest support of 0.002090592 and a lift of 7.358974359. The association rules for the likelihood of LBW are presented in Table 3.

**Table 3:** Association rules among the LR-identified risk factors.

SN	Rules
01	{Division = Dhaka, Twin status = Multiple birth} => {LBW = Yes}
02	{Division = Dhaka, Twin status = Multiple birth, Place of delivery = Private} => {LBW = Yes}
03	{Division = Sylhet, Duration of breastfeeding = Not currently breastfeeding, Birth order = 2^nd^, Wealth index = Low} => {LBW = Yes}
04	{Division = Chittagong, Duration of breastfeeding = Not currently breastfeeding, Place of delivery = Home, Wealth index = Low} => {LBW = Yes}
05	{Division = Sylhet, Twin status = Single birth, Duration of breastfeeding = Not currently breastfeeding, Birth order = 2^nd^} => {LBW = Yes}
06	{Division = Chittagong, Twin status = Single birth, Duration of breastfeeding = Not currently breastfeeding, Place of delivery = Home, Wealth index = Low} => {LBW = Yes}

**Rule 1:** This rule indicates that in the Dhaka division, multiple births are more likely to have LBW. Recognizing this high-risk group can help policymakers and healthcare providers prioritize targeted interventions, such as enhanced prenatal care and nutritional support for mothers expecting multiples in Dhaka. Such focused strategies could reduce LBW incidence and improve neonatal outcomes in this region.

**Rule 2**: This rule demonstrates that in the Dhaka division, multiple births delivered in private healthcare facilities are associated with a higher likelihood of LBW. This insight can inform targeted healthcare policies by emphasizing the need for enhanced prenatal care and specialized monitoring for multiple pregnancies, especially in private sector settings in Dhaka.

**Rule 3**: This rule indicates that in the Sylhet division, 2^nd^ born babies from low-wealth families who are not currently breastfeeding have a higher likelihood of being LBW. This finding suggests the need for targeted nutritional and maternal support programs focusing on vulnerable households in Sylhet, particularly for families with multiple births and those with limited economic resources.

**Rule 4**: This rule indicates that in the Chittagong division, infants from low-wealth households who are delivered at home and are not currently breastfeeding are at a higher risk of LBW. This synergetic cluster can guide targeted healthcare policies and interventions in Chittagong, such as promoting facility-based deliveries, enhancing maternal nutrition support among low-income families, and providing breastfeeding counseling to improve newborn health outcomes in this region.

**Rule 5**: This rule illustrates that in the Sylhet division, single births that are 2^nd^ born babies and are not currently breastfeeding have a higher likelihood of being born with LBW. This rule suggests that targeted interventions in Sylhet could focus on enhanced prenatal care and nutritional support for mothers carrying 2^nd^ born babies, as well as continued breastfeeding practice to improve infants’ postnatal care. Health policies could prioritize resource allocation and awareness campaigns in Sylhet to address specific risk factors and reduce LBW prevalence.

**Rule 6**: This rule shows that in the Chittagong division, single-born babies who are not currently breastfeeding, born at home, and from low-income households are at higher risk of LBW. Understanding this pattern can help guide targeted healthcare interventions, such as promoting facility-based deliveries, supporting breastfeeding programs, and providing nutritional assistance to low-income families, making the findings more actionable and relevant for health policy planning in Chittagong.

## 4. Discussion

This study investigates risk factors and examines their co-occurrence patterns contributing to LBW among Bangladeshi infants, using BDHS 2022 data. LR analysis was applied to identify significant individual risk factors for LBW, while ARM was used to uncover co-occurrence patterns among these risk factors. The LR showed that division, twin status, wealth index, place of delivery, duration of breastfeeding, and birth order are significantly associated with individual risk factors of LBW. The ARM revealed that infants in the Dhaka division with multiple births exhibited a higher risk of LBW, and this risk is also increased when delivery occurs at a private institution. In Sylhet, LBW is more likely among 2^nd^ born children from low-wealth households who are not currently breastfeeding, illustrating the interactive effect of socioeconomic and maternal care factors. In Chittagong, infants from single births who are not currently breastfeeding, delivered at home, and from low-wealth households are also at elevated risk.

Across all regions, lack of breastfeeding and low household wealth repeatedly appear as co-occurring factors, indicating that both maternal behaviors and socioeconomic conditions play a key role in influencing LBW risk. These findings highlight the implications for maternal and child health in Bangladesh by emphasizing the multifaceted interactions among individual, socioeconomic, and regional factors that contribute to LBW. Although breastfeeding saves children’s lives, particularly among vulnerable populations such as low birth weight (LBW) neonates, this relationship should not be interpreted as breastfeeding influencing birth weight [38]. Breastfeeding cannot causally affect birth weight, as it occurs after birth. Infants with LBW are less likely to be breastfed due to immaturity, illness, and separation from their mothers and are often too small or medically fragile to initiate breastfeeding immediately [39]. Studies have shown that LBW infants frequently experience delayed initiation and reduced ability to suckle due to underdeveloped reflexes and medical complications [40–42]. Therefore, the observed relationship likely reflects reverse causality, in which LBW contributes to reduced breastfeeding initiation rather than breastfeeding affecting birth weight. Continued breastfeeding, however, remains critical for improving survival and growth outcomes among LBW neonates once they are clinically stable. Infants born as multiple births, 2^nd^ born babies, or those not currently breastfeeding are at higher individual risk, suggesting the need for enhanced maternal care and postnatal care support.

Improving access to skilled maternal healthcare, supporting maternal nutrition, and encouraging facility-based deliveries are critical for reducing LBW prevalence. Strengthening ANC by ensuring early and regular check-ups, nutrition counseling, and monitoring high-risk pregnancies can prevent LBW, particularly among multiple births and second-born babies. Maternal nutrition programs, including supplementation with iron, folic acid, and other essential nutrients, are essential for fetal growth. Providing breastfeeding support for mothers who have ceased breastfeeding can further improve neonatal outcomes.

Regional disparities observed in divisions such as Dhaka, Sylhet, and Chittagong reflect differences in healthcare infrastructure, cultural practices, and maternal access to services. The higher LBW risk in Dhaka is due to the large proportion of its population living in slums and informal settlements, where poor sanitation, overcrowding, and limited access to quality maternal healthcare increase vulnerability. Poor maternal nutrition, driven by food insecurity and high living costs, contributes to fetal growth restriction. Many women in low-income urban areas engage in physically demanding work, such as in a garment factory or domestic labor, often continuing late into pregnancy, thereby increasing both physical strain and stress. Additionally, environmental factors, especially Dhaka’s high air pollution levels, have been linked to LBW in Bangladesh [43].

In Sylhet and Chittagong, parts of the population live in rural or remote areas with limited healthcare infrastructure, longer travel times to facilities, and lower antenatal service utilization. Regional cultural practices, including home deliveries and variations in breastfeeding, also contribute to LBW risk. The findings also highlight the critical importance of addressing socioeconomic disparities, as low-wealth families are at significantly higher risk of having LBW infants. Low-income household mothers often have limited access to quality healthcare, including antenatal care, skilled birth attendants, and emergency obstetric services, which can delay the detection and management of pregnancy complications [44]. Poor maternal nutrition is another key contributor, as families with lower income may struggle to afford adequate food, supplements, or micronutrients essential for healthy fetal growth.

Additionally, low-wealth households are more likely to experience suboptimal living conditions, such as overcrowding, poor sanitation, and exposure to infections, which can adversely affect maternal and fetal health. From a regional perspective, similar patterns are observed across South Asia, emphasizing that these determinants are not unique to Bangladesh. In India, studies in states like Uttar Pradesh, Bihar, and Odisha have found that low maternal socioeconomic status, multiple births, short birth intervals, home deliveries, and inadequate ANC are major contributors to LBW [45,46]. Likewise, in Nepal, LBW is significantly higher among children born to mothers from low-income households, with poor maternal nutrition and limited access to health facilities [47,48]. The findings of our study demonstrate that the risk factors of LBW in Bangladesh are consistent across South Asia, highlighting shared challenges such as inequality in maternal and child health services, limited nutrition, and reliance on home deliveries.

These identified factors emphasize the need for targeted, multifaceted interventions. Essential strategies include promoting institutional deliveries, strengthening antenatal and postnatal care, improving maternal nutrition programs, supporting breastfeeding, addressing regional healthcare inequities, and assisting low-wealth households. Furthermore, our findings can be translated into practice through healthcare programs by integrating these targeted interventions into maternal and child health services. For example, training midwives or community health workers (CHWs) to identify and monitor high-risk pregnancies, such as those involving multiple births, low socioeconomic status, or suboptimal breastfeeding, can facilitate early intervention.

These frontline workers can be equipped with skills to provide personalized counseling on nutrition, ANC attendance, exclusive breastfeeding, and safe delivery practices. In similar settings, several successful interventions have demonstrated effectiveness in reducing LBW. For example, in Nepal, the Community-Based Newborn Care Program (CB-NCP) trained CHWs to provide home-based counseling and follow-up for pregnant women, leading to improvements in neonatal care practices—particularly increased skin-to-skin thermal care—even though the behavioral impact could not be entirely attributed to the intervention itself [49]. Likewise, Bangladesh’s maternal, neonatal, and child health (MNCH) program, implemented through BRAC, improved facility delivery and early postnatal care through community health volunteers, helping reduce adverse birth outcomes in underserved areas [50].

Drawing on these models, our study recommends integrating targeted risk-factor screening and culturally tailored counselling into existing national maternal health programs. Furthermore, mobile health (mHealth) tools can be introduced to support CHWs in tracking high-risk mothers and delivering timely health messages—particularly given usability challenges observed in existing mHealth apps in Bangladesh [51]. Addressing these factors collectively has the potential to reduce LBW prevalence, improve neonatal survival and health outcomes, and promote equitable maternal and child health, thereby advancing progress toward SDG 3.2 in Bangladesh.

### 4.1. Limitations of the study

The study has several limitations. First, this study used secondary data from the BDHS 2022, which lacked control over variable definitions and data quality. Second, possible biases may arise from self-reported data of the variables, such as birth weight or ANC visits, which are subject to recall or social desirability bias. Third, the unavailability of potentially relevant predictors, such as maternal nutrition, psychosocial stress, and environmental exposures, may have excluded important determinants of LBW. Finally, the cross-sectional design of the study precludes causal inference.

## 5. Conclusion

This study used the BDHS 2022 data to investigate associated individual risk factors using LR and to explore co-occurring patterns among the identified risk factors using ARM. The LR showed that division, twin status, wealth index, place of delivery, duration of breastfeeding, and birth order are significantly associated with individual risk factors of LBW. The ARM revealed these risks were higher when factors occurred together, such as multiple births in Dhaka or low-wealth mothers in Sylhet/Chittagong who were not currently breastfeeding, 2^nd^ birth order, and home delivery. LR effectively identified individual risk factors, while ARM offered more profound insights into how these factors cluster synergistically to increase LBW risk within specific socio-geographic settings. This integration of LR and ARM provides a more comprehensive understanding of both individual and interacting risk factors, underscoring the need for public health interventions that address not only single risk factors but also their interactions. By connecting statistically significant factors to multidimensional vulnerability patterns, this dual approach provides insight that supports the development of more precise, targeted public health strategies to address both universal and locally specific causes of LBW. Such strategies can help reduce LBW prevalence and neonatal mortality in Bangladesh, thereby contributing to achieving Sustainable Development Goal 3 (SDG 3).
